# Hepatocyte Growth Factor-Mediated Chondrocyte Proliferation Induced by Adipose-Derived MSCs from Osteoarthritis Patients and Its Synergistic Enhancement by Hyaluronic Acid

**DOI:** 10.3390/ijms26199296

**Published:** 2025-09-23

**Authors:** Samuel Jaeyoon Won, Hyun-Joo Lee, Dae-Yong Kim, Hyeonjeong Noh, Song yi Lee, Ji Ae Yoo, Yoon Sang Jeon, Heebeom Shin, Dong Jin Ryu

**Affiliations:** 1 Orthopedic Surgery, Inha University Hospital, Incheon 22332, Republic of Korea; samuelwon88@gmail.com (S.J.W.);; 2 School of Medicine, Inha University, Incheon 22013, Republic of Korea; 3 Stem Cell R&D Center, N-BIOTEK, Inc., Bucheon-si 14449, Gyeonggi-do, Republic of Korea; 4 N-BIOTEK, Inc., Bucheon-si 14502, Gyeonggi-do, Republic of Korea

**Keywords:** osteoarthritis, adipose-derived stem cells, spheroids, hepatocyte growth

## Abstract

Mesenchymal stem cells (MSCs) spontaneously assemble into three-dimensional (3D) spheroids under matrix-deficient conditions such as the synovial cavity, although their functional significance has yet to be fully elucidated. In this study, we used concave microwell cultures to promote the spontaneous aggregation of adipose-derived MSCs (ASCs) from OA patients, thereby mimicking the intra-articular microenvironment. We analyzed the paracrine factors of ASC aggregates and compared it with that of conventional 2D monolayer cultures. Notably, 3D aggregation significantly increased the secretion of HGF and VEGF, whereas FGF2 levels remained relatively unchanged. These results indicate that the structural characteristics of ASC aggregates enhance the secretion of key paracrine factors involved in angiogenesis and tissue repair. To functionally evaluate the biological relevance of the secreted factors, conditioned media (CM) from ASC aggregates were applied to human articular chondrocytes. The CM significantly promoted chondrocyte proliferation, an effect that was abolished by the addition of HGF-neutralizing antibodies, thereby highlighting HGF as a central mediator of the regenerative response. Additionally, we further explored whether extracellular factors could modulate growth factor expression such as HGF. In this context, we investigated the impact of low-concentration hyaluronic acid (HA), a key synovial component widely used in OA treatment. Co-treatment with HA not only amplified the expression and secretion of HGF, VEGF, and FGF2, but also promoted ASC proliferation. ASCs forming functional aggregates may exert regenerative effects as active paracrine modulators, and the addition of low-dose hyaluronic acid is expected to further enhance this function, offering a promising strategy for MSC-based osteoarthritis therapy.

## 1. Introduction

Mesenchymal stem cells (MSCs) are emerging as potential candidates for treating various diseases due to their capacity to differentiate into multiple lineages and their immunomodulatory and anti-inflammatory properties [1,2,3]. Among them, adipose-derived MSCs (AD-MSCs) are especially suitable for degenerative joint disorders like osteoarthritis (OA), owing to their accessibility and superior chondrogenic potential [2,4]. Beyond simple differentiation, MSCs secrete various growth factors, anti-inflammatory molecules, and immunomodulators that improve the microenvironment of damaged tissue [1,5,6].

Intra-articular (IA) injection of MSCs is a simple and minimally invasive therapeutic approach that has gained clinical popularity [7,8,9,10,11]. Preclinical studies have demonstrated that IA-administered MSCs facilitate structural recovery of cartilage, suppress cartilage degradation, and promote tissue repair [12,13]. Clinical trials have shown that IA MSCs can reduce pain, improve joint function, and stimulate cartilage regeneration [8,9,10,11,14,15]. However, variability in therapeutic responses and unclear mechanisms of action still present major challenges in standardizing MSC-based therapies [16,17].

Once administered into the joint cavity, MSCs exist in a suspension without extracellular matrix attachment, which encourages them to spontaneously form three-dimensional aggregates (spheroids) [18,19]. These spheroids are not merely cellular clusters—they display unique physiological properties such as enhanced viability, increased resistance to hypoxia, and elevated secretion of regenerative factors [20,21]. Studies show that aggregated MSCs enhance cartilage repair more effectively than monolayer MSCs, likely due to improved paracrine activity [20,21,22]. Thus, developing in vitro 3D spheroid models that mimic intra-articular MSC behavior is critical to understanding their therapeutic effects.

In parallel, hyaluronic acid (HA) is widely used in the treatment of OA due to its lubricative and biomechanical cushioning properties, and it plays a crucial role in maintaining joint homeostasis [23]. As a major component of synovial fluid, HA is known to regulate cell migration, proliferation, and interactions with the extracellular matrix [24,25]. Several recent studies have suggested that HA can enhance the survival, proliferation, and secretory functions of MSCs, highlighting its potential as a biological adjuvant [26,27]. However, the specific mechanisms underlying the combined use of HA and MSCs in OA treatment, particularly in relation to growth factor secretion and therapeutic function, remain unclear.

In this study, we aimed to investigate the effects of intra-articularly injected ASCs on OA patients, particularly focusing on their behavior as cellular aggregates within joint tissue. We sought to elucidate how these aggregates contribute to tissue regeneration and modulation of the joint microenvironment. Furthermore, we evaluated whether co-administration with HA could enhance the paracrine activity and therapeutic efficacy of ASCs.

## 2. Results

### 2.1. Phenotypic Characterization of ASCs and Spheroid Viability

Based on previous reports that MSCs, including ASCs, spontaneously form three-dimensional (3D) aggregates when administered into matrix-deficient environments such as the synovial cavity [14,15], we sought to characterize the biological features of such aggregates in vitro. To this end, autologous ASCs were isolated from three patients with OA. Detailed patient demographics and clinical information are presented in Table 1. To evaluate the intrinsic capacity of ASCs to form 3D aggregates, we utilized a microwell-based culture platform that promotes spontaneous spheroid formation under matrix-deficient conditions.

**Figure 1:**
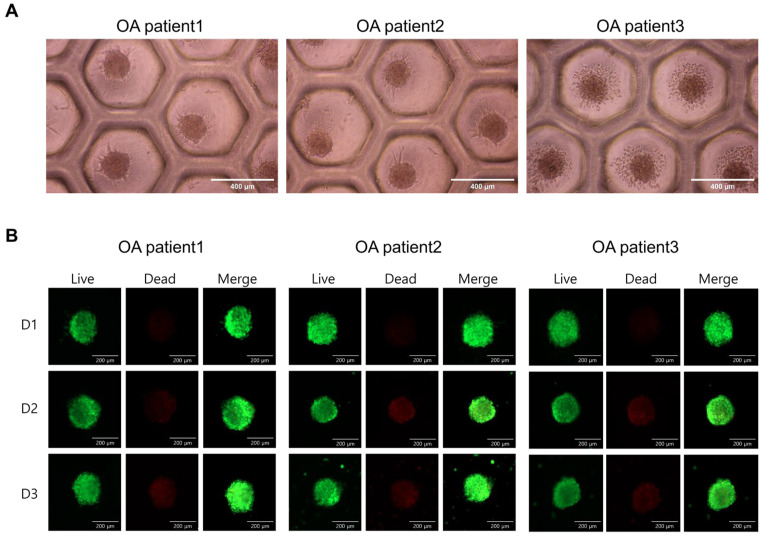
Formation and viability of ASC spheroids derived from osteoarthritis patients. (**A**) Representative bright-field images showing the formation of uniform spheroids by ASCs from three osteoarthritis patients (OA patient 1, 2, and 3) after 3D culture using concave microwell plates. All spheroids exhibit consistent morphology and stable aggregation. Scale bars = 400 μm. (**B**) Live/Dead staining of spheroids at day one (D1), day two (D2), and day three (D3) post-aggregation. Live cells were stained green with calcein-AM, while dead cells were stained red with ethidium homodimer-1. Merged images show predominantly green fluorescence, indicating high viability of the spheroids across all patients and time points. Scale bars = 200 μm.

ASCs were seeded into commercially available concave microwell plates, enabling uniform aggregation without the use of exogenous scaffolding materials. Over a three-day culture period, the cells rapidly self-assembled into compact spheroids, reaching an average diameter of approximately 200 μm by day three. Bright-field microscopy revealed consistent spheroid formation across all three donors, with aggregates displaying a spherical and well-defined morphology, suggesting that ASCs inherently possess aggregation potential when exposed to 3D confinement (Figure 1A).

To further assess the structural stability and metabolic activity of these spheroids, we performed Live/Dead staining using the calcein-AM/ethidium homodimer-1 dual fluorescence assay. Across all time points (day one to day three), the spheroids demonstrated high viability, as evidenced by the strong calcein-AM fluorescence (green) and minimal red fluorescence from ethidium homodimer-1, which stains nuclei of compromised or dead cells. Fluorescence microscopy confirmed that the majority of cells remained viable throughout the culture period, with live cells being evenly distributed throughout the spheroid mass (Figure 1B). Notably, the aggregates maintained their structural integrity without central necrosis or disintegration, which is often observed in larger or less-oxygenated spheroids. This indicates the preservation of spheroid morphology over time, along with sustained cellular viability.

These results indicate that ASCs survive and adapt well under hypoxic and matrix-deprived conditions mimicking the intra-articular environment. ASC spheroids exhibited consistent viability and uniform morphology, characteristics that allowed us to proceed with evaluating regenerative factor secretion and therapeutic potential in osteoarthritis.

### 2.2. Growth-Factor Profile of Spheroid-Conditioned Medium

Having confirmed that ASCs form viable and morphologically uniform spheroids under hypoxic and matrix-deprived conditions, we next sought to examine whether 3D culture modulates the secretion of growth factors. To assess the influence of 3D culture on the secretion of regenerative cytokines, we quantified the concentrations of FGF2, VEGF, and HGF in the conditioned media collected from both 2D monolayer and 3D spheroid cultures of ASCs obtained from three individual patients.

**Figure 2:**
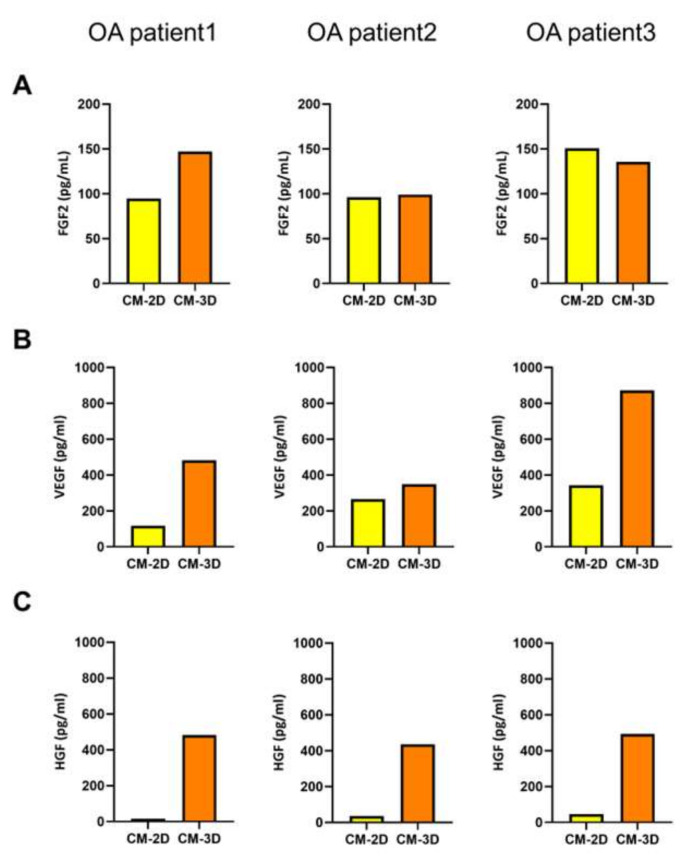
Comparison of FGF2, VEGF, and HGF levels in conditioned medium from 2D and 3D Cultures of ASCs. CM were collected from ASCs derived from three individual OA patients, cultured either as 2D monolayers (CM-2D) or as 3D aggregates (CM-3D). The concentrations of FGF2 (**A**), VEGF (**B**), and HGF (**C**) were measured. FGF2 levels showed only modest differences between 2D and 3D conditions across all patient samples. VEGF levels were consistently increased in CM-3D compared to CM-2D, suggesting enhanced angiogenic potential under 3D culture. HGF secretion was markedly upregulated in CM-3D, exhibiting a 10- to 30-fold increase compared to CM-2D in all patients, indicating that ASC aggregation significantly enhances the secretion of this regenerative cytokine.

The levels of FGF2 in the conditioned media (CM) showed minimal differences between 2D and 3D cultures across all donors. Although a slight increase in FGF2 secretion was observed in 3D conditions, the variation was not consistent or substantial, suggesting that FGF2 production may not be significantly influenced by cellular aggregation or the 3D microenvironment (Figure 2A). In contrast, VEGF levels were consistently elevated in 3D-CM compared to 2D across all patient-derived samples. The extent of the increase varied between donors, but the overall trend indicated a two- to three-fold elevation in VEGF concentration under 3D culture conditions (Figure 2B). Most notably, HGF levels exhibited a dramatic increase in the 3D culture condition. In all three ASC cultures, HGF secretion in the 3D-CM was significantly elevated, showing approximately 10- to 30-fold higher concentrations compared to 2D-CM (Figure 2C). This increase in HGF secretion was consistently observed across all patient-derived samples in 3D spheroid culture.

Collectively, these findings demonstrated that 3D aggregation of ASCs increased the secretion of HGF and VEGF, while having minimal effect on FGF2 expression.

### 2.3. Three-Dimensional-Conditioned Medium Promotes Chondrocyte Proliferation More Effectively than 2D-Conditioned Medium

**Figure 3:**
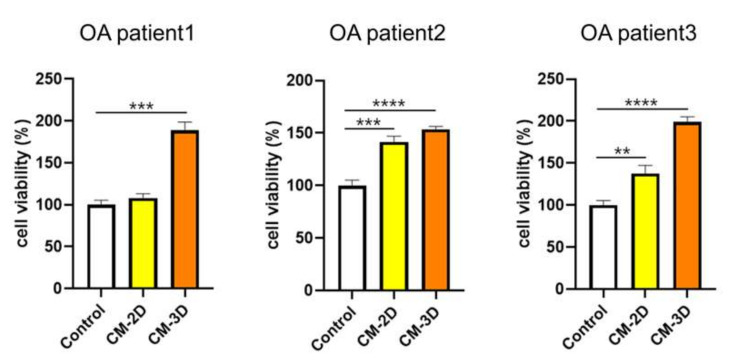
Chondrocyte viability following treatment with conditioned media (CM) derived from 2D or 3D cultures of ASCs from three individual patients. Chondrocytes were cultured for 24 h in either control media (a 1:1 mixture of DMEM and α-MEM), conditioned media collected from ASCs cultured in CM-2D, or conditioned media collected from ASCs cultured as CM-3D. Both CM-2D and CM-3D were mixed at a 1:1 ratio with DMEM prior to treatment to maintain consistent nutrient conditions. CM-3D significantly enhanced chondrocyte proliferation in all three patient-derived samples, showing the greatest increase compared to other treatment groups. Data are presented as the mean ± SD. ** *p* < 0.01, *** *p*< 0.001, ****p < 0.0001.

Given the markedly elevated HGF levels in CM-3D, we next investigated whether this translated into a functional proliferative effect on chondrocytes. Since HGF has been reported to stimulate chondrocyte proliferation and extracellular matrix synthesis through c-Met-dependent signaling pathways [28], we hypothesized that CM-3D would exert stronger trophic effects on chondrocytes than CM-2D. To further evaluate the chondrocyte-proliferative effect of ASCs-derived conditioned media, we analyzed the cell viability of chondrocytes treated with CM-2D and CM-3D derived from three individual OA patients (Figure 3). Across all patient-derived CM samples, CM-3D consistently induced a significant increase in chondrocyte viability compared to both the control and CM-2D groups. For OA patient 1, CM-3D treatment resulted in a statistically significant enhancement of cell viability (*p* < 0.001) compared to control and CM-2D. Similarly, in OA patient 2, both CM-2D (*p* < 0.001) and CM-3D (*p* < 0.0001) significantly increased chondrocyte viability relative to control, with CM-3D showing the greatest effect. In OA patient 3, the trend was also observed, where CM-2D induced a moderate increase (*p* < 0.01), while CM-3D further amplified cell viability with high significance (*p* < 0.0001).

These results indicated that 3D ASC spheroid-conditioned media (CM-3D) exerted a superior pro-proliferative effect on chondrocytes.

### 2.4. HGF Neutralization Reduces the Chondrocyte-Proliferative Effect of 3D ASC-Conditioned Medium

**Figure 4:**
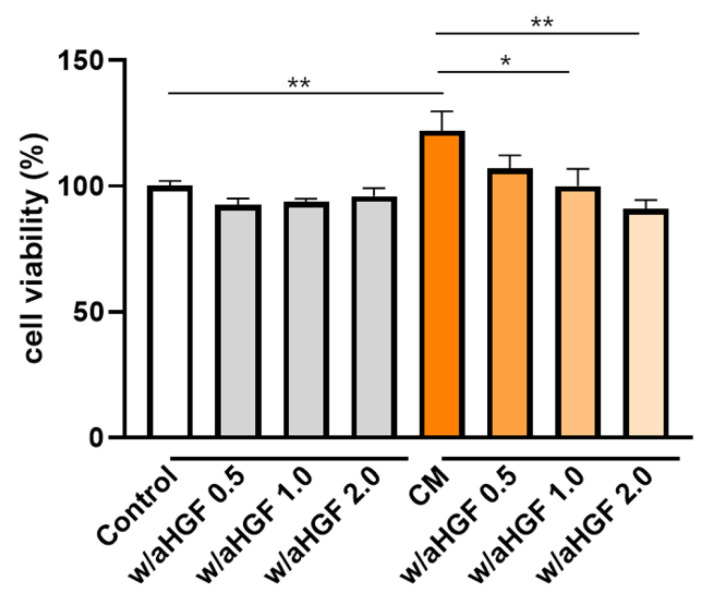
Neutralization of HGF attenuates the proliferative effect of 3D ASC-conditioned medium (CM) on chondrocytes. Chondrocytes were treated with CM alone or CM with anti-HGF antibodies at various concentrations (0.5, 1.0, and 2.0 μg/mL). Cell viability was measured using the CCK-8 assay after 24 h of treatment. CM significantly enhanced chondrocyte proliferation, whereas HGF neutralization suppressed this effect in a dose-dependent manner. Data are presented as mean ± SD. * *p* < 0.05, ** *p* < 0.01.

Given the markedly elevated HGF levels observed in CM-3D, we sought to determine whether this factor directly mediates the observed proliferative effects on chondrocytes. To validate the role of HGF in mediating chondrocyte proliferation induced by CM-3D, a neutralization assay using anti-HGF antibodies was conducted. As shown in Figure 4, CM alone significantly enhanced chondrocyte viability compared to the control (*p* < 0.01). However, when anti-HGF antibodies were co-administered with the CM, cell viability decreased in a dose-dependent manner. Notably, treatment with 1.0 μg/mL and 2.0 μg/mL of anti-HGF significantly reduced the proliferation-enhancing effect of the CM (*p* < 0.05 and *p* < 0.01, respectively), while 0.5 μg/mL showed a modest reduction that was not statistically significant.

### 2.5. HA Modulates Growth-Factor Secretion in 30 Patient-Derived ASCs

MSCs combined with HA have been reported to exert synergistic effects in tissue regeneration, enhancing differentiation, cell viability, migration, and anti-inflammatory responses [26,27]. Previous studies have shown that HA can influence the paracrine profile of MSCs through interactions with surface receptors such as CD44, thereby enhancing the secretion of angiogenic and chondrogenic factors [29,30,31].

We next examined whether HA could further enhance ASCs functionality by modulating the secretion of regenerative growth factors such as HGF. To examine this, ASCs obtained from 30 OA patients were cultured in the presence of HA. HA was mixed with ASCs at two concentrations, a high concentration of 100 µg/mL and a low concentration of 10 µg/mL, and changes in the expression of growth factors according to these concentrations were analyzed. As shown in Supplementary Data S2, treatment with 10 µg/mL HA markedly increased the secretion of HGF and FGF2 in the majority of donors compared to untreated controls, whereas 100 µg/mL HA showed variable effects on VEGF production across donors.

**Figure 5:**
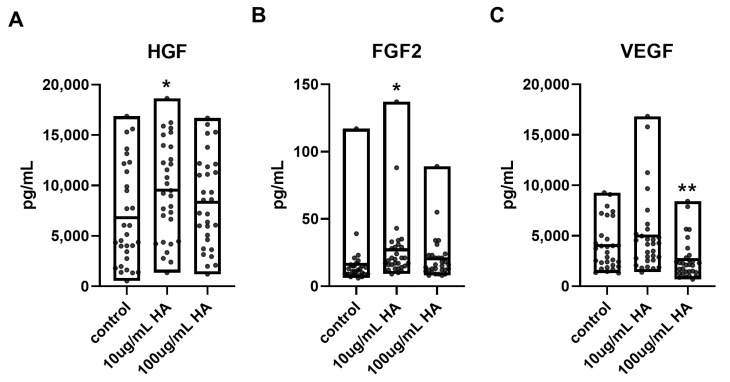
Hyaluronic acid (HA)-induced modulation of growth factor secretion in OA patient-derived ASCs. ASCs were cultured under three different conditions: untreated control (control), low-concentration hyaluronic acid (10 µg/mL), and high-concentration hyaluronic acid (100 µg/mL). The secretion levels of (**A**) HGF, (**B**) FGF2, and (**C**) VEGF from 30 OA patient-derived ASC cultures. HGF secretion was significantly increased under 10 µg/mL HA (*p* < 0.01) compared to both non-treated and 100 µg/mL HA groups. FGF2 also showed significant elevation with 10 µg/mL HA (*p* < 0.05), while high concentration HA did not enhance expression. VEGF expression was significantly decreased in the 100 µg/mL HA group compared to both control and 10 µg/mL HA conditions (*p* < 0.05), suggesting a concentration-dependent inhibitory effect. The results are shown as the mean ± SD. * *p* < 0.05, ** *p* < 0.001.

Overall, HGF expression levels were elevated in HA-treated groups compared to untreated controls. Notably, 10 µg/mL HA induced higher HGF expression than 100 µg/mL HA in 20 out of 30 patients. Analysis of FGF2 and VEGF expression showed a comparable trend: 10 µg/mL HA resulted in increased FGF2 levels in 20 patient samples, and VEGF in 16 patients, compared to high HA treatment. However, statistical analysis revealed that while HGF and FGF2 expression levels were significantly higher under low HA conditions, VEGF showed a significant decrease in the high-concentration HA group (Figure 5).

These results indicated that low-dose HA enhances the regenerative paracrine activity of ASCs more effectively than high-dose HA, particularly for HGF and FGF2, which are critical for chondrogenesis and cartilage tissue repair.

### 2.6. HA Enhances ASC Proliferation in a Dose-Dependent Manner

**Figure 6:**
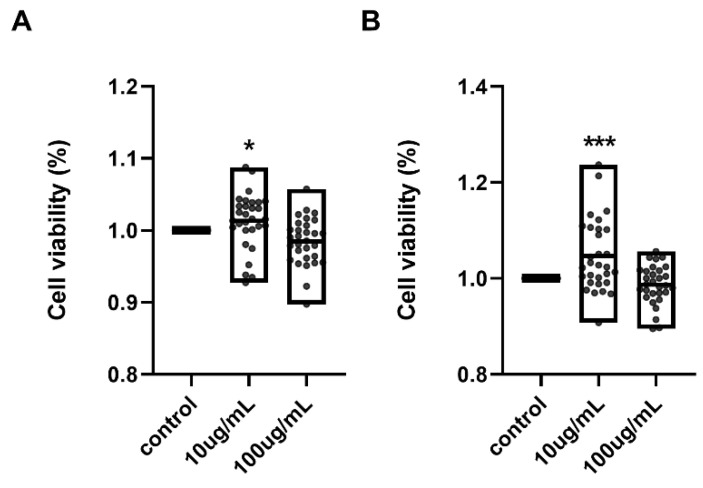
Effect of hyaluronic acid concentration on proliferation of ASCs. ASCs were cultured under the same three conditions described in Figure 5. Cell proliferation was assessed using the Cell Counting Kit-8 (CCK-8) assay at 24 h (**A**) and 72 h (**B**) after treatment. Each condition was tested in triplicate, and absorbance at 450 nm was measured to quantify viable cells. Cells treated with 10 µg/mL HA exhibited significantly higher proliferation rates compared to the control and 100 µg/mL HA groups at both time points. In contrast, 100 µg/mL HA treatment showed either no significant difference or a slight decrease in proliferation. The results are shown as the mean ± SD. * *p* < 0.05, *** *p*< 0.001.

To further examine the effect of HA on the proliferative capacity of ASCs, we conducted a cell proliferation assay at 24 and 72 h following HA treatment. ASCs derived from 30 individual OA patients were cultured with either low-concentration HA (10 µg/mL), high-concentration HA (100 µg/mL), or without HA, as a control. Low-concentration HA treatment led to a significant enhancement in cell proliferation in all donor-derived samples, compared to both the untreated and high-concentration HA groups (*p* < 0.05 at 24 h; *p* < 0.001 at 72 h) (Figure 6A). Conversely, cells treated with high-concentration HA showed no significant difference in proliferation compared to controls at 24 h, and a slight but statistically non-significant decrease at 72 h. These results indicated that the proliferative response of ASCs to HA treatment was concentration-dependent, with low-dose HA enhancing proliferation, while high-dose HA did not confer such an effect.

## 3. Discussion

MSCs have garnered attention due to their immunomodulatory properties, regenerative potential, and capacity to secrete trophic factors that modulate the local tissue microenvironment [31,32]. In OA, the intra-articular injection of MSCs represents a promising therapeutic modality, especially given their relative abundance and ease of harvest from patients [33,34,35].

Importantly, MSCs injected into matrix-deficient environments—such as the synovial cavity—tend to spontaneously aggregate, forming spheroid-like structures in vivo. This behavior is thought to be physiologically relevant, as aggregation enhances cell viability, sustains stemness, and upregulates key regenerative factors via hypoxia-inducible pathways and cell–cell interactions [36,37,38,39]. Consistent with this, our study employed ASCs to form 3D spheroids in vitro, mimicking this in vivo aggregation process. These spheroids exhibited high viability and robust secretion of HGF and VEGF confirming their potential as dynamic trophic units that could modulate cartilage repair processes [40,41,42]. Among the factors secreted, HGF played a particularly important role in stimulating chondrocyte proliferation, a finding that aligns with prior reports describing its chondrogenic, anti-apoptotic, and matrix-preserving effects [43,44,45]. Neutralization of HGF significantly impaired the proliferative capacity of the spheroid-conditioned medium, suggesting that HGF is a critical effector molecule mediating ASC-induced regeneration.

In this study, we further examined the effects of HA on ASC viability and secretory activity to explore its potential clinical applications. Although HA has been widely used for its joint-lubricating effects in osteoarthritis treatment [23,46], emerging evidence indicates that its interaction with MSCs may enhance therapeutic outcomes through synergistic mechanisms. Moreover, HA is increasingly recognized as a bioactive molecule capable of modulating MSC function through receptor-mediated mechanisms [47,48,49,50]. Specifically, interactions between HA and CD44 or RHAMM receptors can trigger intracellular signaling cascades, including PI3K/AKT and MAPK/ERK, which regulate cell survival, proliferation, and trophic factor secretion [51,52,53]. Our findings show that low concentrations of HA (10 µg/mL) significantly enhanced the proliferation and growth factor secretion of ASCs, whereas higher concentrations (100 µg/mL) had no such effect and even suppressed the expression of certain factors. This biphasic response is consistent with previous findings indicating that excessive concentrations of HA can increase the viscosity of the extracellular environment, thereby impeding receptor–ligand interactions and reducing cellular responsiveness and downstream signaling. Furthermore, high-molecular-weight or concentrated HA has been shown to impair cell adhesion, proliferation, and differentiation due to steric hindrance or altered integrin-mediated signaling [54,55,56], highlighting the importance of optimizing HA concentration for favorable cell–matrix interactions. 

Interestingly, previous studies have shown that engineered 3D encapsulation systems, such as HA/alginate core–shell microcapsules, enhance VEGF and PDGF secretion from MSC spheroids compared to non-encapsulated controls. Compared to these studies, which utilized engineered 3D scaffolds, our study analyzed the effects of HA on ASCs cultured in 2D monolayers, focusing on how HA influences trophic factor secretion and proliferation prior to spheroid formation. This approach allows us to better understand the priming effect of HA on ASCs under simpler culture conditions, which could complement 3D aggregation studies and inform optimized clinical applications.

Nonetheless, this study has several limitations that should be considered when interpreting the findings. First, all experiments were conducted in vitro under simplified 2D culture conditions, which do not fully recapitulate the complex and dynamic joint microenvironment. The joint environment in vivo is highly complex, characterized by dynamic mechanical loading, immune surveillance, and biochemical gradients that can significantly influence the behavior and therapeutic efficacy of transplanted cells [57,58]. In vivo, implanted cells are exposed to mechanical loading, hypoxia, immune surveillance, and inflammatory cytokines (e.g., IL-1β, TNF-α), all of which can significantly influence ASC survival, the stability of aggregate, and trophic factor secretion [59,60]. Therefore, in vivo validation using OA animal models is essential to evaluate whether the enhanced HGF secretion observed in this study translates into meaningful cartilage regeneration and functional recovery. We are currently designing in vivo studies using OA animal models to assess the therapeutic efficacy of co-administered HA and ASCs. These studies will focus on histological analysis of cartilage repair, modulation of inflammatory markers, and long-term functional outcomes. The investigations are critical for translating our in vitro findings into clinically relevant strategies and optimizing ASC-based therapies for osteoarthritis.

While this study focused exclusively on ADSCs, it is increasingly recognized that MSCs represent a highly heterogeneous population composed of multiple subpopulations with distinct functional capacities [61]. Emerging evidence highlights skeletal stem cells (SSCs) as a more defined and functionally homogeneous subpopulation with superior chondrogenic and osteogenic potential compared to conventional MSCs [62,63]. In addition, other stem cell sources—such as bone marrow-derived MSCs (BM-MSCs), umbilical cord-derived MSCs (UC-MSCs), and synovium-derived stem cells (SDSCs)—have demonstrated variable regenerative properties, differentiation potentials, and clinical applicability [64,65,66]. Given these differences, the optimal selection of stem cell sources or combinations tailored to patient-specific conditions may be critical to achieving maximal therapeutic efficacy in osteoarthritis treatment. Future studies should therefore focus on comparative analyses of these distinct stem cell populations to establish evidence-based strategies for selecting the most suitable seed cells for cartilage repair.

In conclusion, HA not only acts as a mechanical scaffold but also as a biochemical modulator that enhances the secretory potential of ASCs. When combined with 3D spheroid formation, this approach significantly boosts the paracrine-mediated regenerative capacity of ASCs, particularly through HGF secretion. These findings provide a strong rationale for optimizing ASC-based OA therapies by optimizing the HA microenvironment and exploiting the natural aggregation behavior of ASCs.

## 4. Materials and Methods

### 4.1. Patient Recruitment and Ethics

This procedure was performed as a preparatory step for the intra-articular administration of ASCs in patients with knee OA. After obtaining informed consent, adipose tissue was collected from the abdominal region of 30 patients diagnosed with knee OA (Kellgren–Lawrence grade II: 12 patients, grade III: 12 patients, grade IV: 6 patients). Local anesthesia was administered to the periumbilical skin using 5 mL of 1% lidocaine (Huons, Sungnam-si, Gyeonggi-do, Republic of Korea). Subsequently, a tumescent solution composed of 250 mL of 0.9% normal saline (JW Medicine, Gwacheon-si, Gyeonggi-do, Republic of Korea), 20 mL of 1% lidocaine (Huons), 1 mL of 1:1000 epinephrine (DAI HAN PHARM CO, Seoul, Republic of Korea), and 5 mL of 8.4% sodium bicarbonate (Huons) was infiltrated into the subcutaneous fat layer to ensure uniform dispersion. After a 10 min waiting period to allow tissue emulsification, a liposuction cannula was inserted through a small 0.7 cm skin incision into the subcutaneous fat, and suction was applied at a pressure of 150 mmHg. Careful handling was performed to minimize bleeding, and approximately 70 to 100 mL of aspirate—including blood and tumescent fluid—was obtained. The procedure was conducted by a single orthopedic surgeon (DJR). The study protocol received approval from the Institutional Review Board of Inha University Hospital (IRB number 2023-06-020).

### 4.2. Isolation and Expansion of Osteoarthritis-Adipose-Derived Mesenchymal Stem Cells

ASCs were isolated from adipose tissue of OA patients collected at INHA University Hospital. The harvested adipose tissue was washed three times with phosphate-buffered saline (PBS) (Lonza, Bassel, Swizerland) and enzymatically digested using 0.1% (*w*/*v*) Type I collagenase (Nordmark Pharma GmbH, Uetersen, Germany) at 37 °C for 45 min. After digestion, undigested tissue fragments and oil were removed by passing the suspension through a 100 μm mesh filter, followed by centrifugation at 1500 rpm for 5 min. The resulting cell pellet was resuspended in primary culture medium composed of α-Minimum Essential Medium (α-MEM) (Welgene, Gyeongsan-si, Gyeongsangbuk-do, Republic of Korea) supplemented with 1% fetal bovine serum (FBS) (Gibco, New York, NY, USA). Cells were plated at a density of 1 × 10^4^ cells/cm^2^ in fresh medium and cultured in a humidified incubator at 37 °C with 5% CO_2_. After 24 h, non-adherent cells were removed by replacing the medium with complete culture medium consisting of α-MEM (Welgene), 9% FBS, and 1 ng/mL basic fibroblast growth factor (bFGF) (R&D Systems, Minnneapolis, MN, USA). The culture medium was refreshed every 2 to 3 days thereafter.

### 4.3. Flow Cytometric Phenotyping

To characterize the phenotypic identity of ASCs isolated from osteoarthritis (OA) patients, surface antigen profiling was performed using flow cytometry. A representative ASC sample was stained with antibodies against common mesenchymal stem cell (MSC) markers (CD73, CD90, and CD105, Beckman Coulter, Brea, CA, USA), as well as hematopoietic lineage markers (CD45 and CD34, Beckman Coulter), and analyzed to confirm MSC identity. The data are presented in Supplementary Data S1.

### 4.4. Spheroid Formation and Viability Assay

To generate three-dimensional (3D) ASC aggregates, cells were seeded into concave microwell plates (StemFIT 3D^®^, MicroFIT, H389600, Hanam-si, Gyeonggi-do, Republic of Korea) at a density of 1.2 × 10^6^ cells per mold. After 5 h of incubation, the culture medium was carefully replaced to remove non-aggregated cells. Aggregates were maintained for up to 3 days with daily medium changes. As a control, ASCs were cultured in parallel under conventional two-dimensional (2D) monolayer conditions using α-MEM supplemented with 10% fetal bovine serum (FBS), 1 ng/mL bFGF, and 1% penicillin-streptomycin at 37 °C and 5% CO_2_. The morphology and size of the aggregates were observed by light microscopy, and cell viability within the spheroids was assessed using the LIVE/DEAD Viability/Cytotoxicity Kit (Invitrogen™, L3224, Waltham, MA, USA). Live cells were stained with calcein-AM (green fluorescence), while dead cells were stained with ethidium homodimer-1 (red fluorescence). Aggregates were then imaged under a fluorescence microscope to assess viability and structural integrity.

### 4.5. Collection of Conditioned Medium (CM) and Growth-Factor Quantification

ASCs were cultured under both two-dimensional (2D) monolayer and three-dimensional (3D) spheroid conditions using identical culture media (α-MEM supplemented with 10% FBS, 1 ng/mL of bFGF, 1% penicillin-streptomycin) at 37 °C in a 5% CO_2_ atmosphere. For 3D spheroid cultures, conditioned medium was collected daily immediately before media change, taking care to avoid aspiration of cells or spheroids. Collected culture media were transferred into 1.5 mL tubes and centrifuged at 300× *g* for 5 min to remove floating cells and debris. The supernatant was filtered through a 0.4 μm filter, aliquoted, and stored at −80 °C until analysis. Conditioned media from 2D cultures were collected and processed using the same procedure.

The levels of Hepatocyte Growth Factor (HGF), Fibroblast Growth Factor (FGF), and Vascular Endothelial Growth Factor (VEGF) in the conditioned media were quantified using the LEGENDplex multi-analyte flow assay kit (BioLegend, San Diego, CA, USA) according to the manufacturer’s protocol. Standard curves were generated by serial dilution of standard reagents. Fluorescent beads specific to each target growth factor were incubated with samples and standards. Subsequently, samples were mixed with detection antibodies and incubated at room temperature on a digital orbital shaker (N-BIOTEK, Bucheon-si, Gyeonggi-do, Republic of Korea) at 300 rpm for 2 h. After washing three times with wash buffer, detection antibodies conjugated with fluorescent labels were added and incubated for 1 h at 300 rpm. Without washing, Streptavidin-PE (SA-PE) was added and incubated for an additional 30 min at 300 rpm. After two final washes, fluorescence intensity was measured using a flow cytometer. Concentrations of growth factors were calculated based on the standard curves using the LEGENDplex™ software, a cloud-based program for flow cytometry data analysis (BioLegend, San Diego, CA, USA; available at: https://www.biolegend.com/en-us/immunoassays/legendplex/support/software, accessed on 11 June 2025).

### 4.6. Evaluation of Chondrocyte Proliferation by Conditioned Media Derived from 2D- and 3D-Cultured ASCs

To evaluate the proliferative effects of conditioned media (CM) derived from ASCs cultured under two different conditions, human articular chondrocytes (TC28a2, Merck Millipore, Burlington, MA, USA) were seeded in 96-well plates (SPL) at a density of 1 × 10^3^ cells per well in 100 μL of culture medium (DMEM high glucose supplemented with 10% FBS and 1% penicillin-streptomycin). CM was collected from conventional 2D monolayer cultures (CM-2D) and 3D spheroid cultures generated using concave microwell plates (CM-3D). After 24 h of incubation at 37 °C in a 5% CO_2_ atmosphere, the medium was replaced according to the experimental conditions. For the treatment groups, 50 μL of previously collected CM-2D or CM-3D was mixed with 50 μL of fresh DMEM (10% FBS, 1% penicillin-streptomycin) and added to each well. For the control group, 50 μL of DMEM (10% FBS, 1% penicillin-streptomycin) was combined with 50 μL of α-MEM containing 10% FBS, 0.1–10 ng/mL bFGF, and 1% penicillin-streptomycin. After an additional 24 h incubation, cell proliferation was measured using a CCK-8 assay (DOJINDO, Rockville, MD, USA). Briefly, 10 μL of CCK-8 reagent was added to each well and incubated for 2 h at 37 °C. The absorbance was measured at 450 nm using a microplate reader to quantify cell proliferation.

### 4.7. Neutralization Assay to Evaluate the Role of HGF in Chondrocyte Proliferation

To determine whether HGF mediates the proliferative effect of CM-3D on chondrocytes, a neutralization assay was performed using anti-HGF antibodies. Human articular chondrocytes were seeded at a density of 1 × 10^3^ cells per well in 96-well plates with 100 μL of DMEM (high glucose) supplemented with 10% FBS and 1% penicillin-streptomycin. After 24 h of incubation at 37 °C and 5% CO_2_, the medium was replaced according to experimental conditions. For the CM-treated groups, 50 μL of 3D-CM was mixed with 50 μL of DMEM (10% FBS, 1% penicillin-streptomycin) and added to each well. In neutralization groups, anti-HGF antibodies (Sino Biological, Beijing, China) were added at concentrations of 0.5, 1.0, or 2.0 μg/mL to the CM mixture before application. The control group received 50 μL of DMEM and 50 μL of α-MEM (10% FBS, 1 ng/mL bFGF, and 1% penicillin-streptomycin) supplemented with an equivalent concentration of isotype control antibodies. After an additional 24 h incubation, cell proliferation was evaluated by adding 10 μL of CCK-8 reagent per well, followed by a 2 h incubation. Absorbance was measured at 450 nm to quantify cell viability.

### 4.8. Hyaluronic Acid Dose–Response Study

To investigate the effect of hyaluronic acid (HA) on growth factor expression in ASCs, ASCs were isolated from the subcutaneous adipose tissue of 30 OA patients. The phenotypic characterization of the isolated cells is presented in the Supplementary Figure. Cells were plated and cultured under standard conditions (37 °C, 5% CO_2_) for 24 h to allow for adhesion. After media removal, the cells were cultured in media containing either low-concentration (10 µg/mL) or high-concentration (100 µg/mL) HA. The HA-supplemented media were prepared by diluting Synovian^®^ (20 mg/mL hyaluronic acid; LG Chem, Seoul, Republic of Korea) to achieve final concentrations of 100 µg/mL (direct dilution) and 10 µg/mL (1:10 dilution of the high concentration). Cells were incubated in each HA condition under hypoxic conditions (2% O_2_) for 48 h. After treatment, conditioned media were collected and analyzed using ELISA kits specific for human HGF (R&D Systems, DHG00B), TGF-β1 (DB100C), FGF2 (DFB50), and VEGF (DVE00). All samples were measured in duplicate, and concentrations were normalized to pg/mL. The data are presented in Supplementary Data S2.

### 4.9. Statistical Analysis

All experiments were performed at least in triplicate, and data are expressed as mean ± standard deviation (SD). Differences among three groups were analyzed using one-way analysis of variance (ANOVA) followed by Dunnett’s multiple comparison test to compare each group with the control. All analyses were performed using GraphPad Prism software (version 8.0.2, GraphPad Software, San Diego, CA, USA). Statistical significance was defined as *p* < 0.05.

## Figures and Tables

**Table 1:** Detailed patient demographics and clinical information of three donors.

	OA Patient 1	OA Patient 2	OA Patient 3
Age (years)	62	61	54
Sex	Female	Female	Male
BMI (kg/m^2^)	25.4	23.6	27.1
Smoking	-	-	1 pack/day
Alcohol	-	-	1 bottle/week
K-L grade	II	IV	IV

## Data Availability

The data that support the findings of this study are available upon reasonable request.

## Supplementary Materials

The following supporting information can be downloaded at: https://www.mdpi.com/article/10.3390/ijms26199296/s1.
