# Supplementary material for: Hepatocyte Growth Factor-Mediated Chondrocyte Proliferation Induced by Adipose-Derived MSCs from Osteoarthritis Patients and Its Synergistic Enhancement by Hyaluronic Acid
Source: Int J Mol Sci. 2025 Sep 23;26(19):9296. doi: 10.3390/ijms26199296 (PMC12525082; doi:10.3390/ijms26199296)
Supplement: Supplementary file 1 [file ijms-26-09296-s001.zip › ijms-3796749-supplementary.pdf]

Supplementary Data

Hepatocyte Growth Factor - Mediated Chondrocyte Prolifera-tion Induced by Adipose-Derived MSCs from Osteoarthritis Patients and Its Synergistic Enhancement by Hyaluronic Acid

Samuel Jaeyoon Won 1†, Hyun-Joo Lee 3†, Dae-Yong Kim 3, 4, Hyeonjeong Noh 3, 4, Song yi Lee 3, Yoo Ji Ae 3, Yoon Sang Jeon 1, Ji Hoon Baek 1 and Dong Jin Ryu 1, 2*

[1] Orthopedic Surgery, Inha University Hospital, Incheon 22332, Republic of Korea

[2] School of medicine, Incheon 22013, Republic of Korea

[3] Stem cell R&D center, N- BIOTEK, Inc., 104-706, Technopark Ssangyong 3Cha, 397, Seokcheon-ro, Bucheon-si 14449, Gyeonggi-do, Republic of Korea

[4] N- BIOTEK, Inc., 402-803, Technopark, 655, Pyeongcheon-ro, Bucheon-si 14502, Gyeonggi-do, Republic of Korea

* Correspondence: mdryu24@naver.com

Supplementary data S1

Sample ID/ CD markers	CD34 (%)	CD45(%)	CD73(%)	CD90(%)	CD105(%)
S001	0.13	0.14	100	100	99.99
S002	0.25	1.18	99.89	100	99.99
S003	0.12	0.01	100	100	99.97
S004	0.59	0.44	99.97	99.87	99.95
S005	0.26	0.12	99.99	99.99	99.99
S006	0.28	0.22	99.94	99.98	99.94
S007	0.32	0.32	100	99.99	99.85
S008	0.18	0.13	99.98	99.99	99.96
S009	0.1	0.09	100	99.99	99.96
S010	0.11	0.21	99.99	100	100
S011	0.09	0.24	99.99	100	99.93
S012	0.01	0.18	99.99	99.99	99.99
S013	0.04	0.3	100	100	100
S014	0.57	0.35	99.99	100	99.98
S015	0.02	0.04	100	99.98	99.98
S016	0.11	0.13	100	99.99	100
S017	0.07	0.08	100	100	100
S018	0.16	0.1	99.99	99.98	99.98
S019	0.04	0.08	99.98	100	99.95
S020	0.26	0.2	100	100	99.98
S021	0.07	0.08	99.99	99.99	99.99
S022	0.09	0.07	99.99	99.99	99.98
S023	0.04	0.21	99.98	99.99	99.99
S024	1.61	0.54	99.99	99.98	100
S025	1.11	0.9	96.59	100	99.94
S026	0.08	0.09	99.98	99.95	100
S027	0.16	0.29	99.99	99.97	99.97
S028	0.39	0.95	99.98	99.87	99.98
S029	0.1	0.12	100	99.93	100
S030	0.13	0.12	99.97	99.35	97.79

Supplementary data1. Flow cytometric analysis of mesenchymal stem cell (MSC) surface markers in ADSCs from individual osteoarthritis patient. Flow cytometry was performed to assess the expression levels of canonical MSC surface markers. The percentage of cells positive for CD73, CD90, and CD105 (positive markers), and CD34, CD45 (negative markers) are shown for each patient sample.

Supplementary data S2

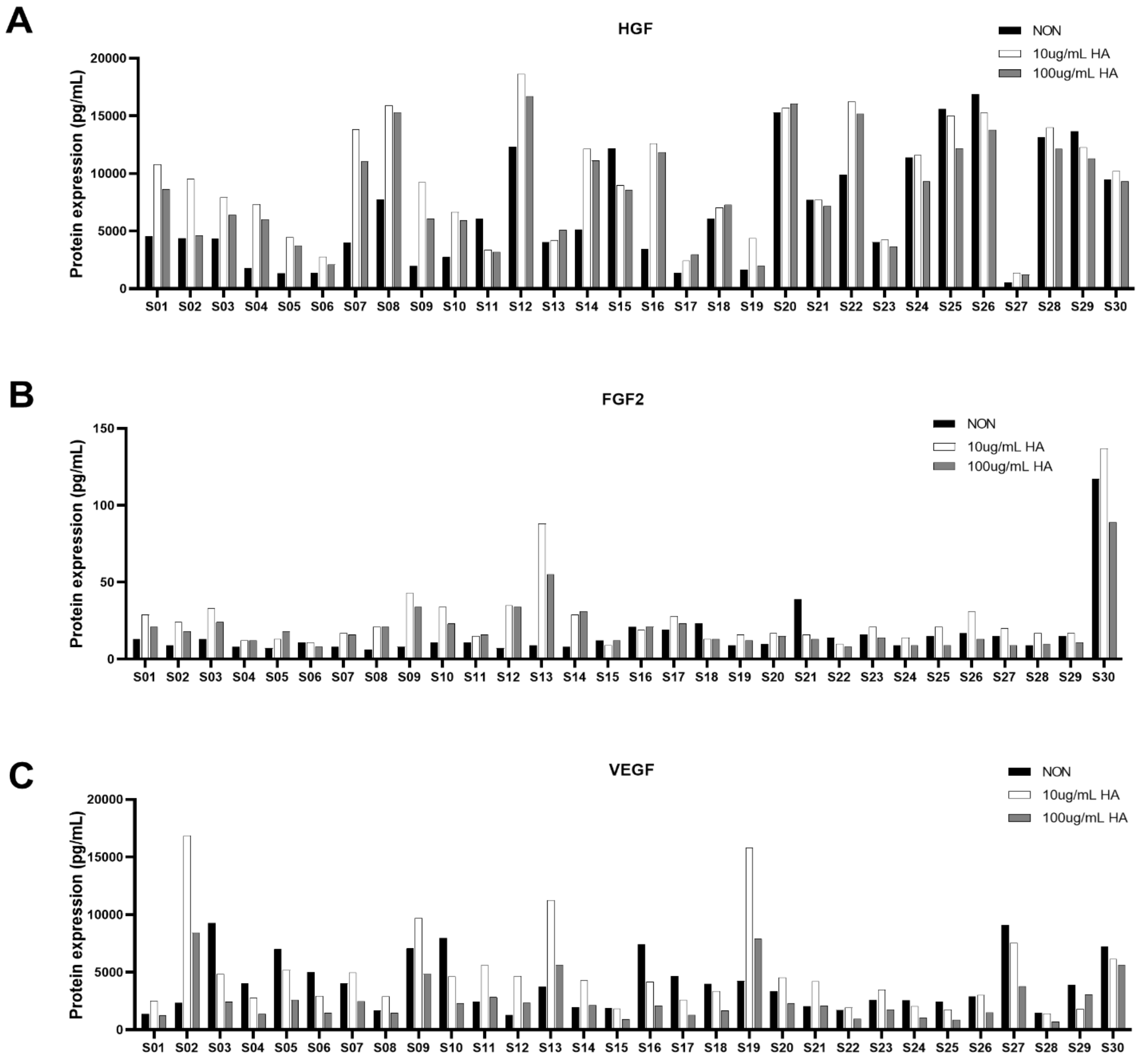

Supplementary data 2. Effect of hyaluronic acid (HA) on the secretion of regenerative growth factors in ASCs derived from osteoarthritis (OA) patients.ASCs obtained from 30 OA patients were cultured with or without HA at two concentrations (10 µg/mL and 100 µg/mL) for 48 hours. The levels of (A) HGF, (B) FGF2, and (C) VEGF in the culture supernatants were quantified using ELISA. Black bars represent untreated control (No HA), white bars indicate treatment with 10 µg/mL HA, and gray bars represent treatment with 100 µg/mL HA. In most donors, 10 µg/mL HA markedly enhanced the secretion of HGF and FGF2, whereas VEGF expression exhibited variable patterns, with several donors showing reduced VEGF levels at 100 µg/mL HA.
